# Fetal Arthrogryposis Secondary to a Giant Maternal Uterine Leiomyoma

**DOI:** 10.1155/2012/726732

**Published:** 2012-11-12

**Authors:** José María Vila-Vives, Juan José Hidalgo-Mora, Inmaculada Soler, Juan Rubio, Ramiro Quiroga, Alfredo Perales

**Affiliations:** ^1^Department of Obstetrics, La Fe University Hospital, Bulevar Sur s/n, 46026 Valencia, Spain; ^2^Department of Obstetrics, Regional Hospital of Vinaroz, Avenida de Gil de Atrocillo s/n, 12500 Castellon, Spain

## Abstract

Arthrogryposis multiplex congenital is a rare condition defined as contractures in multiple joints at birth due to disorders starting in fetal life. Its etiology is associated with many different conditions and in many instances remains unknown. The final common pathway to all of them is decreased fetal movement (fetal akinesia) due to an abnormal intrauterine environment. Causes of decreased fetal movements may be neuropathic abnormalities, abnormalities of connective tissue or muscle, intrauterine vascular compromise, maternal diseases, and space limitations within the uterus. When the cause of arthrogryposis is space limitations in uterus, the most common etiology is oligohydramnios. The same can result from intrauterine tumours as fibroids, although to our knowledge there are only two papers reporting cases of fetal deformities related to uterine leiomyomas. We describe a well-documented exceptional case of arthrogryposis associated with the presence of a large uterine fibroid. It could illustrate the importance of a careful and appropriate assessment of uterine fibroids before and in the course of a pregnancy considering that they can cause both serious maternal and fetal complications.

## 1. Introduction

Arthrogryposis multiplex congenital is a rare nonprogressive condition defined as contractures in multiple joints at birth, due to disorders starting in fetal life. Although antenatally diagnosed, arthrogryposis results often in prenatal death, many children with this disease survive and its overall prevalence is 8.5 per 100,000, without difference in sex ratio [1, 2]. 37% cases had isolated AMC, 12% had additional syndrome or chromosomal anomalies, and 51% had other major malformations [1]. The etiology of arthrogryposis is associated with many different conditions and in many instances remains unknown. Nevertheless, the final common pathway to all of them is decreased fetal movement (fetal akinesia) due to an abnormal intrauterine environment [3]. Causes of decreased fetal movements may be neuropathic abnormalities, muscle abnormalities, abnormalities of connective tissue, intrauterine vascular compromise, maternal diseases, and space limitations within the uterus [2]. 

 When the cause of arthrogryposis is space limitations in uterus, the most common etiology is oligohydramnios, which will limit the movements of the fetus, especially when it occurs early in gestation. The same can result from twin pregnancies, deformities of the uterus, and, theoretically, from intrauterine tumours as fibroids, although nowadays very few cases have been described proving this association [4]. There are some cases described with oligohydramnios, but to our knowledge there are only two papers reporting cases related to uterine leiomyomas [5, 6]. We describe a well-documented rare case of arthrogryposis caused by the presence of a large fibroid. 

## 2. Case Presentation

A 33-year-old pregnant woman was admitted to our department in the 16th week of her first gestation because of suspicion during a routine ultrasound of decreased fetal movements and fetal malformation consisting of postural deformity of both lower limbs associated with a uterine fibroid. The family history was unremarkable and the mother's medical history nonsignificant except for the presence of two intramural uterine myomas diagnosed before the current pregnancy and smaller than 47 mm in maximum diameter in previous control one year before gestation. 

**Figure 1:**
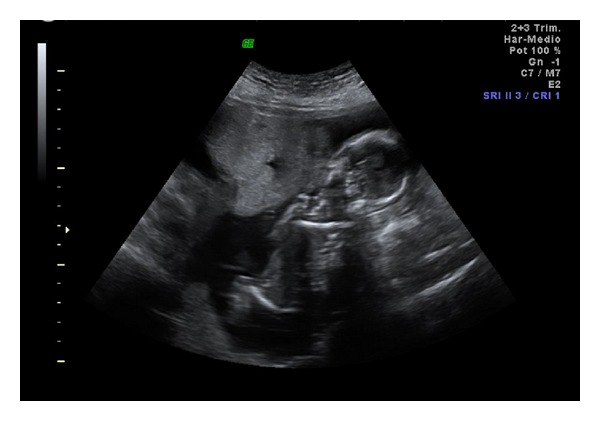
Abdominal ultrasound image showing the myoma in the front face of the uterus that clearly evidences the fetal compression. Both fetal legs and arms are in a hyperflexion position.

 Our ultrasound investigation verified the presence of an intramural-submucosal tumor with a muscular echogenic pattern and a size of 110 × 88 mm in the anterior uterine wall near to the internal cervical os and another intramural one with a size of 28 × 21 mm in the uterine fundus. Fetus presented a normal heart rate pattern and was observed with absent movements and displaced to the right uterine wall in an abnormal position, with hyperextension of head and strongly flexed lower limbs (Figure 1). Fetal measures according to the gestational age and the amount of amniotic fluid were normal. On the basis of these findings, the diagnosis of arthrogryposis secondary to fetal akinesia due to giant leiomyoma was done.

**Figure 2:**
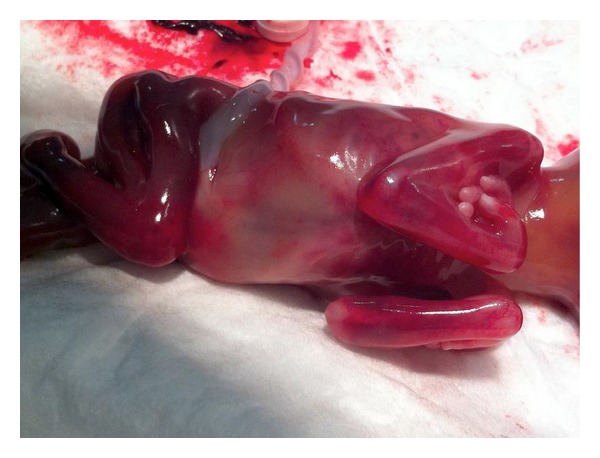
Photography of the fetus showing both legs and arms flexed and abnormally positioned.

According to the Spanish legislation, the parents decided the elective termination of pregnancy. A vaginal delivery was induced with local prostaglandins without complications. At the time of birth, the fetus had not cardiac activity and showed external male genitals, a weight of 105 g and a length of 125 mm. His position consisted of internal rotation of the shoulders and stiffness of the limbs with symmetrically flexed and fixed elbows and wrists and severe equinovarus deformities of the feet (Figure 2).

 The pathology report concluded that internal organs of the fetus did not present abnormalities. Histopathological examination reported extraconnective tissue developed around the joints and tissue between the muscle fibers swollen with the presence of red blood cells. Chromosomal analysis was normal and the study of fetal DNA excluded the spinal muscular atrophy because there were no deletions of the SMN1 gene.

## 3. Discussion

The movement seems to be necessary for normal growth of the limbs and joints during intrauterine life. If the fetus stops moving, the joints become stiff. It is then difficult for them to stretch and resume normal movement in utero [7]. This is the pathogenesis of congenital arthrogryposis. The time point during development at which the limitation of movement begins is probably critical in determining the degree, the type of contractures, and the involvement of other organ systems [3]. The earlier and longer the duration of decreased movements during fetal development, the more severe the contractures will be. Associated with the lack of fetal movement, extraconnective tissue develops around the joints. This fixes the joint in place, limiting the joint movement and aggravating the contractures [2]. In our case, according to the decreased fetal movement due to space limitation in uterus, this connective tissue in joints was the more significant histopathological finding, in addition to the edema between muscle fibers in limbs.

 Congenital arthrogryposis usually occurs as a sporadic event, but a proportion of cases have a genetic origin involving autosomal dominant, recessive, or X-ligated mechanisms, being a component of a number of genetic syndromes. So it is important to try to determine a specific diagnosis with the objective of establishing the mode of inheritance and risk of recurrence for the purpose of counseling family members and also in following the natural history, which may be quite variable within a family [2, 3]. 

 Prenatal ultrasound diagnosis of arthrogryposis is focused on diminished fetal movements and the presence of fixed articular contractures and abnormal positioning of extremities. Nevertheless, these signs are very difficult to detect during the first trimester of pregnancy. For this reason, multiple congenital contractures are usually diagnosed during the second trimester. Ultrasound diagnosis of them is based on observation of scarce or absent fetal movements, which should lead to careful examination of the joints and detection of the anomalies that allow prenatal diagnosis. When the joints of the upper limbs are affected, the shoulders are usually in internal rotation, the forearms are pronated, and there is congenital wrist and radius dislocation with flexion of the hands in a fixed position. If the legs are affected, there may be hip flexion with congenital dislocation. The knee joints may be closed to each other in a hyperextended position and the feet are in varus position. The classical image is that of strongly flexed hands, pes equinovarus and the fetus in a Buddha-like position [8]. Prenatal ultrasound can also be used to identify associated abnormalities. The lungs are the most frequent site of involvement, besides the limbs. In utero, the fetus has respiratory movements, which are necessary for normal development of the lungs. If the fetus has not moved much, the lungs may be hypoplastic, which is the most common cause of intrauterine and neonatal elevated mortality with congenital contractures [7].

 Space restriction in uterus, mainly, secondary to oligohydramnios, has been reported as a cause of fetal deformities, but very few cases related to the presence of leiomyomas have been described until now. Leiomyomas of the uterus are detectable in approximately 2% of pregnancies and cause complications in the course of pregnancy or delivery in 1 of 10 diagnosed cases. These complications include premature rupture of membranes, premature birth, placental abruption, necrosis, postpartal hemorrhage, puerperal sepsis, compression on the maternal organs, fetal malpresentation, and increased incidence of abortions and cesarean sections [9]. Less well known are the potential fetal complications associated with special restriction of the uterine cavity caused by uterine leiomyomas, causing limb reduction, caudal dysplasia, and head deformation and congenital torticollis [10]. The size and location of the fibroids accompanying a pregnancy have been reported as the most important factors causing these complications. Concerning changes in size during pregnancy, although there are significant differences between scientific papers, some of these papers describe a great increase in volume of myomas during the first trimester of gestation [10, 11]. Probably this occurred in our case and the previous 47 mm fibroid in anterior uterine wall grow-up quickly during the year before and, specially, in the course of the first trimester of pregnancy protruding into the uterine cavity. 

 The medical literature generally advocates conservative therapy for leiomyomas during pregnancy. Nevertheless, it is important to be aware of the possibility of complications and to consider myomectomy prior to the gestation because the growth of the leiomyoma cannot be predicted. Even, surgical intervention in pregnant patients should be assessed because, in addition to classical complications, spatial restrictions could have a deleterious effect on human embryos and fetuses.
